# Fattening Pig Farmers’ Intention to Participate in Animal Welfare Programs

**DOI:** 10.3390/ani9121042

**Published:** 2019-11-28

**Authors:** Sirkka Schukat, Alina Kuhlmann, Heinke Heise

**Affiliations:** Agribusiness Management, Department of Agricultural Economics and Rural Development, University of Göttingen, 37073 Göttingen, Germanyheinke.heise@agr.uni-goettingen.de (H.H.)

**Keywords:** animal welfare, initiative animal welfare, unified theory of acceptance and use of technology

## Abstract

**Simple Summary:**

The demand for products with higher farm animal welfare (FAW) standards is increasing within the European Union. In response, representatives from agricultural associations, the slaughtering industry and food retailing started the industry solution Initiative Animal Welfare (IAW) in 2015 with the aim of establishing higher FAW standards on a broad basis in both poultry and pig production in Germany. In contrast to other available animal welfare programs (AWPs), the IAW receives considerable support from farmers and enjoys a large number of participants. This article focuses on the question why the IAW is gaining positive resonance among German livestock farmers. Applying partial least squares path modeling to analyze the determinants of farmers’ intention to participate in the IAW, we found that the determinants performance expectancy, social influence, facilitating conditions, hedonic motivation, price value, trust and experiences with the IAW have a significant influence on the behavioral intention to participate in the IAW program. It is shown that the farmers’ hedonic motivation has the greatest impact on participation. The prospect of improving the image of pig fattening and the economic incentive motivate many farmers to participate. Additionally, many farmers confirmed that they enjoy providing a more superior welfare status to their pigs. The social determinant was also identified as a strong influencing factor. The results suggest that participation in AWPs could be improved by appropriate remuneration and measures promoting farmers’ intrinsic motivation to engage in AWPs.

**Abstract:**

Farmers are considered a highly important stakeholder group for the successful implementation of higher farm animal welfare (FAW) standards, but so far little is known about their attitudes and the determinants of their participation in programs that request higher FAW standards. To close this research gap, fattening pig farmers in Germany were questioned via a large-scale online survey in 2018 (*n* = 239). Based on the Unified Theory of Acceptance and Use of Technology, a partial least squares path modeling (PLS) was run. Results show that the expected performance as well as the expected costs associated with the Initiative Animal Welfare (IAW) substantially influence fattening pig farmers’ behavioral intention to participate in the IAW. Furthermore, the decision is influenced by social determinants and facilitating conditions such as deadweight effects. Farmers’ hedonic motivation, fair remuneration and previous experiences with the establishment of higher FAW standards can influence their intention to take part in the IAW. In addition, farmers’ trust in the program is a major determinant. There are also moderating variables such as age and work experience that influence farmers’ intention to take part in the IAW. Our results have important managerial implications for the IAW and can help to design further tailor-made animal welfare programs (AWPs) that fulfill the requirements of both fattening pig farmers and the broader public not only in Germany but the European Union.

## 1. Introduction

The modern keeping of farm animals like pig fattening holds a high potential for social conflict in modern Western societies. Concerns about animal welfare and ethics criticisms have increased significantly in recent years [1]. Amongst other things, this results in changes in animal ethological research. Animal welfare is no longer solely assessed against the background of husbandry systems and management measures, but animal health and animal behavior are increasingly becoming the focus of attention [2]. Above all, consumers and politicians are demanding higher animal welfare standards in modern agriculture [3,4]. In response to growing public discourse and to improve farm animal welfare (FAW), various animal welfare programs (AWPs) have been introduced in several EU member states. In Germany, for instance, the Initiative Animal Welfare (IAW) was founded in 2015. It aims at a general improvement of FAW in German poultry and pig production, whereas farmers are compensated by the food retail sector itself for implementing additional FAW criteria. 

The IAW aims at achieving an improvement in FAW in order to meet consumer demands and advanced transparency in meat production. Poultry and pig production are included. The concept stipulates that farmers are rewarded for the implementation of certain FAW criteria on their farms [5]. Recent consumer surveys show that the concept of the IAW is rated “very good” to “good” by 93% of the respondents [6]. In Germany, 10.7 million fattening pigs are already kept in accordance with IAW criteria. This reflects the positive attitude and willingness of farmers to participate. This corresponds to approximately 22% of fattening pigs produced in Germany. Participating food retailers, e.g., Aldi, Edeka, Kaufland, Lidl and Rewe, pay an additional payment of 6.25 cents to the IAW per kilo of pork or poultry meat sold, thus contributing to the costs incurred by farmers in implementing the IAW [6]. As a result, food retailers invest around €130 million per year, with 94.5% of the budget being used to reward the farmers. The remaining budget is spent for communication, internal business processes of the IAW organization and farm audits. Since 2018, a total of 4024 pig fattening farms have participated in the IAW [7]. Table 1 gives an overview of the applicable basic, mandatory and eligible criteria in pig fattening. Quality and Safety is a quality assurance system that covers all stages of production and trade of meat and meat products from the farmers to the shop counters.

For compliance with the basic criteria, a yearly lump sum of €500 per farm is paid. In addition, the participating farmers receive €3.30 per slaughter pig for the implementation of the mandatory criteria. Furthermore, eligibility criteria can also be applied voluntarily. In order to meet the IAW participation requirements, all basic criteria must be complied with. These include, among others, animal monitoring and care, handling of diseased and injured animals as well as hygiene and storage of animal feed. In addition, annual stable climate and drinking water controls must be executed by IAW experts. The climate control includes a functional test of the technology and a sensory pen climate examination. At the drinking water check, physiochemical and microbiological examinations are carried out. In addition, the pens must have access to daylight. The mandatory criteria include “additional 10% space” and “organic manipulable material” which could, for example, constitute wood or straw. If the farm chooses an eligibility criterion like for example “additional 20% space”, the remuneration is granted in addition to remuneration for compliance with the mandatory criterion “additional 10% space”. The maximum fee is €5.10 per pig, which means the eligible criteria cannot exceed €1.80 per pig [8].

Of course, there are international approaches to implement FAW through AWPs. The Dutch animal welfare label “Beter Leven” consists of a three-step system in which the products are marked with one, two or three stars. Depending on the number of stars, higher animal welfare standards are implemented on the participating farms. The animal protection label “Coop Naturafarm” from Switzerland offers animal products that are produced in accordance with special guidelines, animal-friendly and at national level. The “Global Animal Partnership” initiative from the USA also offers a six stage catalogue of criteria for the implementation of FAW, which focuses on animal health, a natural husbandry environment and the emotional well-being of the animals. All programs take place at the national level. No information can be found on the remuneration of farmers in any of the three international programs. Overall, the international approaches are difficult to compare with the IAW, as they have little transparency regarding farmers’ remuneration and the criteria are partly inaccurately defined. 

Since farmers are the ones who have to actively implement animal welfare measures, FAW depends to a considerable extent on their acceptance of FAW and their willingness to implement active measures within the framework of AWPs. So far, there has been no empirical study that refers exclusively to the participation determinants of farmers within the pig fattening sector. To close this research gap, this article aims to identify the influencing factors that determine farmers’ intentions to participate in the IAW. A prior study already showed that the introduction of the IAW is largely supported by farmers [9]. The identification of the determinants shall help to understand why many fattening pig farmers participate in the IAW and how even more farmers can be persuaded to participate in AWPs. Without the understanding of the farmers’ decisions for or against certain measures, FAW can hardly be improved [9]. Of course, also consumers and policy play an important role in this debate [10]. Nevertheless, establishing a higher level of FAW depends, above all, on the farmers’ acceptance for implementing FAW criteria [11]. To achieve this aim, farmers were surveyed about their attitudes on FAWs and AWPs, especially the IAW. The data were then analyzed using the Unified Theory of Acceptance and Use of Technology 2 (UTAUT 2) to evaluate which determinants affect the participation of farmers in FAW programs such as the IAW.

## 2. Conceptual Framework

The UTAUT 2 model is used to investigate the farmers’ acceptance of the IAW and the factors that determine intention to participate. This model is an extended version of the UTAUT which was introduced as a conceptual basis for investigating decisionmakers’ intention to use or not to use a technology or system-such as the IAW [12]. It contains seven key constructs (performance expectancy, effort expectancy, social influence, facilitating conditions, hedonic motivation, price value and habit) that determine behavioral intention to use a technology [12]. In the development of UTAUT 2, some moderating influences were identified such as age, gender and experience [12]. In the course of a literature research and due to the results of previous studies, further constructs were added to the original UTAUT 2 model in order to adapt the model to the research question of this study.

**Figure 1:**
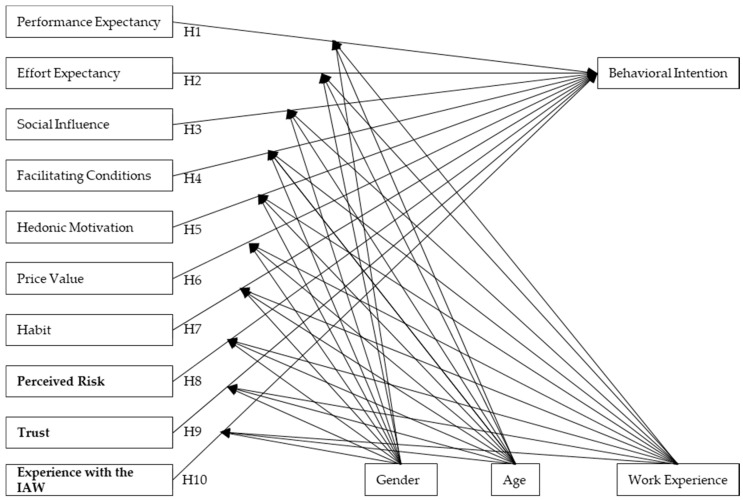
Model used in the partial least squares (PLS) analysis according to UTAUT 2 [12].

Nearly 25% of non-participants say they are basically ready to attend the IAW. Due to the high willingness to participate in the first round, only 49% of the farmers who wanted to participate in the IAW could due to a limited access because of budget constraints. The other farmers were put on a waiting list [13]. It may be that farmers, who generally want to participate, cannot yet. Thus, the actual participation depended on factors other than just the acceptance of the farmers. For this reason, we suspect a distortion of the results, which is why the original construct use behavior, which describes the actual participation of farmers in the IAW, was excluded from the model. Figure 1 summarizes all hypotheses of this study in one model. The three constructs which were added in order to adjust the UTAUT 2 model to this study are highlighted in bold letters.

The behavioral intention involves the intent or non-intention of participating in the IAW. It reflects the attitude of farmers towards IAW participation, with some influence of moderators on the strength of relationships between independent variables and behavioral intention. A multigroup analysis (MGA) is used to examine the moderating effects of age, gender and work experience. MGA offers the possibility to divide a moderator into groups and to examine the influence continuously throughout the model [14]. The moderator work experience in this case is the farmer’s professional experience. It is believed that work experience could have a moderating impact on participation, as experienced farmers may have different views from those who are inexperienced. The determinants are explained in the following and hypotheses shall be derived.

Performance expectancy is defined as the degree of expectation an individual believes that the use of a system, in this case the IAW, is beneficial and brings support and relative advantage. The construct is often understood to be the strongest influencer of behavioral intention [11]. Gender and age seem to play a moderating role in the relationship between performance expectancy and behavioral intention. Studies on gender differences show that young men are more performance orientated than women [11]. In agriculture, the economic performance plays a significant role in the adoption of technologies or systems. Economic aspects are also one of the major reasons for farmers to participate in AWPs [8]. However, some farmers also see the improvement of housing conditions and FAW as a benefit of AWPs [15]. Many farmers regard FAW as important for their animals’ health and thus for their individual performance [16]. For this reason, the following hypotheses can be derived for this study:

**Hypothesis** **1** **(H1).** *Performance expectancy has a significant influence on behavioral intention.*

**Hypothesis** **1a** **(H1a).** *Gender, age and work experience have an impact on the relationship between performance expectancy and behavioral intention.*

The effort expectancy determinant is understood as the expected effort in using a system, with the expected effort often being perceived as higher in a new system at an early stage. Effort includes both financial aspects and time. It is expected that older women with little work experience value the effort higher than men. Therefore, gender, age and work experience play a moderating role on the relationship between effort expectancy and behavioral intention [11]. The perceived effort to use a new system increases with age, as the time required for the learning process in order to use a system is higher [17]. The additional effort involved in participating in AWPs is critically assessed by many farmers. In particular, they mention temporal stress during documentation and control audits as well as restrictions in their daily work at the farm [15] Another study found that the willingness to participate in AWPs decreases as the implementation effort increases [18]. Based on these statements, it is reasonable to suppose that the effort expectancy influences the behavioral intention. From this, the following hypotheses for the work can be derived:

**Hypothesis** **2** **(H2).** *The effort expectancy of the farmers has an influence on the behavioral intention to participate in the IAW.*

**Hypothesis** **2a** **(H2a).** *The effect of effort expectancy on behavioral intention is moderated by age, gender and work experience.*

Social influence on behavioral intention is determined by important persons, such as friends, colleagues and relatives, who influence the individual in using a system or technology. This construct is moderated by age, gender and work experience [11]. Social pressure, which emanates from the media or politics, is also considered. This could also be a reason why pig and poultry farmers in particular are willing to participate in AWPs [19]. In a study, the social impact of colleagues, friends and family on strategic farm decisions was studied [20]. It was found that topics such as corporate development, decent agriculture and the adaption of nature conservation are associated with social influence. The social environment, such as friends and family, influence the operational development of the farm to a certain extent. In addition, it was found that the experience of a farmer’s neighbor with new technologies has a significant impact on future use [21]. Family members also have great influence on strategic decisions for the farm’s development [22]. The following hypotheses are derived:

**Hypothesis** **3** **(H3).** *Social influence affects the behavioral intention to participate in the IAW*.

**Hypothesis** **3a** **(H3a).** *The effect of social influence on behavioral intention is moderated by age, gender and work experience*.

The construct facilitating conditions describes to which degree the respondents believe that an organizational or technical infrastructure exists on the farm that facilitates the use of the system. The facilitating conditions have both influence on the behavioral intention and the adoption itself [11]. These influences are moderated by age, gender and work experience. Many farmers participate in the IAW due to deadweight effects. Measures that are particularly easy to implement on farm level are most often realized by farmers [8]. It is likely that farmers will be more willing to participate in AWPs if they already have the necessary infrastructure on their farm and need to invest less in FAW. From this, the following hypotheses for this study are derived:

**Hypothesis** **4** **(H4).** *Facilitating conditions have a positive influence on the intention to participate in the IAW.*

**Hypothesis** **4a** **(H4a).** *The relationship between behavioral intention and facilitating conditions is moderated by age, gender and work experience.*

Hedonic motivation is defined as the pleasure that arises by using the system. It describes the respondent’s motivation to use a system or technology. Hedonic motivation is moderated by age, gender and work experience [12]. In a further study it was noted that the main motivation for participation is often the financial incentive [23]. The same finding was also made during a survey among pig farmers in Europe [15]. It was indicated that participation in AWPs is often motivated by reasons such as maintaining productivity and profitability [24]. Some farmers see their motivation as an opportunity to provide their animals with more natural housing conditions. Ecological farmers were also motivated by their moral attitude to improve FAW [15]. In addition, many farmers see participation in the IAW as an opportunity to improve the image of conventional pig farming [8]. The following hypotheses are established:

**Hypothesis** **5** **(H5).** *Hedonic motivation significantly influences the behavioral intention to participate in the IAW.*

**Hypothesis** **5a** **(H5a).** *The relationship between motivation and behavioral intention is moderated by age, gender and work experience.*

Price value describes the trade-off between the benefits of using a system or technology and the monetary costs of using it. If the perceived benefit exceeds the monetary costs, then the respondent is more willing to use the system. In case of this, the price value has a positive influence on the behavioral intention to participate in the IAW. The impact of price value is moderated by age, gender and work experience [12]. Farmers run a business with their farm and must generate profit for their businesses in order to remain competitive [22]. The price value that farmers receive for participation is an important factor from the farmers’ perspective. In general, farmers are open to change in form of FAW measures, as long as they receive adequate compensation [8]. It was found that 60% of the surveyed pig farmers are willing to improve FAW if they earn as much as in their current economy [25]. The following hypotheses are set for the study:

**Hypothesis** **6** **(H6).** *Price value has a significant influence on the behavioral intention to participate in the IAW.*

**Hypothesis** **6a** **(H6a).** *The relationship between price value and behavioral intention is moderated by age, gender and work experience.*

Habit describes the extent to which the respondents learn to perform behaviors automatically. The experience of the interviewees also plays a role here. In this case, habit means how quickly and easily the IAW can be integrated into the organizational processes of the farm and into the farmer’s existing habits. Habit has a positive influence on both the behavioral intention and the adoption itself. These relationships are moderated by age, gender and work experience [12]. The following hypotheses are derived:

**Hypothesis** **7** **(H7).** *Habit has an influence on the behavioral intention to participate in the IAW.*

**Hypothesis** **7a** **(H7a).** *The relationship between behavioral intention and habit is moderated by age, gender and work experience.*

To adjust the UTAUT 2 model to this study’s issue, three more constructs were added to the model: perceived risk, trust and experience with the IAW. The background for these considerations is a qualitative survey among farmers on the IAW [8]. Again, these constructs are believed to moderate the strength of the relationship between the constructs and the behavioral intention of age, gender and work experience.

Some studies on farmers’ attitudes towards AWPs show that risk perception plays an important role. Farmers fear the risk of participating in AWPs regarding to economic risk, especially since FAW products are only niche products with low market demand. They doubt that consumers are permanently willing to spend more on these products. They also fear losing their independence when participating in AWPs [15,23]. In addition, many farmers express distrust of the economic benefits of participation. The unannounced control audits in the German IAW, for instance, increase the additional stress and risk of participation from the farmers’ point of view [26]. In 2018, 959 farms did not successfully pass the control audits and lost their remuneration [7]. For this reason, the construct “perceived risk” is added to the model as another possible determinant of intention to participate. It is defined as the perceived risk of both financial disadvantages and perceived stress associated with the use of the system. In a study it was shown that men are more willing to take risks than women which declines with increasing age [27]. From this, it can be assumed that gender and age could have a moderation effect. A possible moderated effect of work experience is also expected. The following hypotheses are derived:

**Hypothesis** **8** **(H8).** *Perceived risk has as significant influence on the behavioral intention to participate in the IAW.*

**Hypothesis** **8a** **(H8a).** *The relationship between perceived risk and behavioral intention is moderated by age, gender and experience.*

Trust is an important determinant for a successful business relationship in the meat industry [28]. In a study it was noted that many measures in AWPs are not accepted by farmers, who believe that the measures are not beneficial to the animals’ health [15]. Due to the large number of potential participants in 2015 not all farmers were able to actually participate in IAW due to budget constraints. These farmers were displeased because they had already invested in FAW measures [8]. In addition, the IAW reduced the maximum remuneration and the catalog of criteria for pig fattening in 2018 [5]. Although, as a consequence, more farmers could participate, they now receive less money if they want to maximize the remuneration [29]. This could have a negative impact on farmers’ trust. Therefore, trust has been added as a further construct to the original model. In a study, it was discovered that women often have more trust than men and that trust decreases with increasing age [30]. The moderation effect of work experience is also investigated. Taken the above together, the study hypothesizes:

**Hypothesis** **9** **(H9).** *Trust influences the intention to participate in the IAW.*

**Hypothesis** **9a** **(H9a).** *The effect of trust in behavioral intention is moderated by age, gender and work experience.*

A further construct added to the model is the experience with the IAW. The assumption was made that experiences that were already made with the IAW exert an influence on farmers’ behavioral intention. Earlier negative or positive experiences can influence the behavioral intention to join a FAW program and thus the actual adoption [15]. It is expected that the relationship also has moderating effects. Therefore, the following hypotheses are put forward:

**Hypothesis** **10** **(H10).** *Experience with the IAW influences the intention to participate in the IAW.*

**Hypothesis** **10a** **(H10a).** *The relationship between experience with the IAW and behavioral intention is moderated by age, gender and work experience.*

## 3. Material and Methods

### 3.1. Sampling and Analysis Methods

For this article, a survey is analyzed in which 239 conventional fattening pig farmers in Germany participated from February 2018 to June 2018. The survey was based on an anonymous online questionnaire. The survey was distributed via the IAW as well as on the homepage of leading German farm management magazines and the Association of Pig Farmers in Germany. In addition, Chamber of Agriculture was targeted. To further spread the link to the survey, private networks and social media were used. The survey questions mainly consisted of closed questions which had to be answered on five-point Likert scales. Further, it was divided into two parts. In part A, the farmers were asked about socio-demographic and farm characteristics, for example age, gender, agricultural education, work experience, farm size, pig housing conditions and participation in the IAW. Part B consisted of 70 statements that were used to determine the farmers’ attitudes towards IAW participation. They were asked to rate the given statements on the basis of five-point Likert scales from “completely agree” to “completely disagree”. The statements should have been answered by both IAW participants and non-participants. The data serve as indicators of the constructs analyzed in the UTAUT 2 model. 

For the analysis, the adjusted UTAUT 2 model was used. The constructs of the model, whose relation to behavioral intention is to be investigated, are so-called latent variables. Latent variables are not directly observable and therefore have to be describes by means of empirical indicators [31]. In this work, covariance structure analyses including the partial least squares (PLS) analysis are used. With PLS analysis, relationships of latent variables, which are the determinants of the intention to participate in the IAW, are determined in the form of the extended UTAUT 2 model. Furthermore, it examines how the latent variables can be described by indicators or manifest variables [31]. In a reflective measurement model as presented in this paper, it is assumed that the indicators are caused by the latent variables. The indicators are linked to the associated latent variables by a simple regression. Finally, the model examines the relationships between the latent variables. A distinction is made between endogenous dependent and exogenous independent variables [32]. Latent variables that affect other latent variables are called exogenous variables. On the other hand, latent variables that have at least one causal relationship are termed endogenous [33]. In this case, only behavioral intention is a dependent endogenous variable. To conduct the PLS analysis, the SmartPLS 3.2.8 analysis software was used. IBM SPSS Statistics 25 software was used for the descriptive analysis of the questionnaire.

### 3.2. Quality Criteria of the Measurement and Structural Model

To check the quality of the measurement model, the so-called indicator reliability must first be controlled. This states whether an indicator is sufficient to describe the construct. The value of the indicator loadings on each construct should be at least 0.6 but better 0.7. A value of 0.7 ensures that at least 50% of the variance of the indicator is explained by the corresponding construct [34]. These will be explained below. In order to check whether the set of indicators sufficiently describes the construct, the Cronbach’s alpha values are determined [35]. The values should ideally exceed 0.7 [36]. To verify the convergence criteria, the average variance extracted (AVE) of the indicators is recorded for the respective construct and the general construct reliability. The AVE describes the variance that is shared by the construct and its indicators [37]. Values of 0.6 or more represent good construct reliability [38]. The AVE value should at least exceed 0.5 [37].

To investigate the discriminant validity of the measurement model, the Fornell–Larcker criterion must first be met. This is the case, if the squared correlations of the variables are smaller than the AVE [39]. In order to achieve discriminant validity, the cross-loadings of the indicators are also checked. An indicator must have a significantly higher loading on its own construct than on others [37]. If a cross-loading, for example, the loading on an unassigned construct is too high, the indicator must be removed. These two criteria are not sufficient to guarantee the discriminatory validity of the measurement model [40]. In order to fulfill these criteria, the heterotrait/monotrait ratio (HTMT) of the correlations must be controlled. The HTMT of the correlations should not exceed 0.85 [40]. Table 2 gives an overview of the quality criteria for the measurement model.

After the validity and reliability of the measurement model has been sufficiently achieved, the structural model itself is checked. The structural model is an illustration of the possible factors influencing the variable that is to be explained and, thus, examines the relationships between the variables [14]. To check the structural model, several steps must be performed. First, the determinate R² of the endogenous variable and the predictive validity, which can be determined by Stone-Geisser’s Q², are examined [41,42]. The extent and significance of the path coefficients are also determined [37]. The determinate measure of R² of the endogenous variable determines the proportion of the explained variance and measures the quality of the fit of the regression function to the indicators [43]. The interpretation is identical to traditional regression analysis. The larger R², the higher the proportion of the explained variance [37]. To some social scientists, a value of R² higher than 0.25 is acceptable [44]. This ensures that at least 25% of the variance of the endogenous variable is explained by the influencing factors. The path coefficients of the structural model can be interpreted like the beta coefficients of a regression analysis. The coefficients are checked for significance by t-statistics. Here, resampling methods are used [33]. In the present paper, the path coefficients’ significance is determined by means of the non-parametric bootstrapping method. The bootstrapping method creates subsamples that are randomly pulled out of the record. These are covered before each new path. These subsamples are used to estimate the PLS path model. The parameter estimates (for example loadings and path coefficients) of the subsamples are used to calculate the standard deviation. This results in the t-values for the significances of the parameters [37,45]. The path coefficients should exceed a value of 0.2 in order to be considered as a meaningful influencing factor [37]. Others say path coefficients are accepted as low as 0.1 [46]. The predictive validity of the model can be determined using the Stone–Geisser test [41,42]. The test is determined by means of the so called “blind following” procedure, which systematically assumes that part of the raw data matrix is missing during parameter estimation [33]. The procedure removes data and treats it as missing values in the parameter estimation. This shows how well the data can be reconstructed by the model. A Q² > 0 implied that the model has good predictive validity [37].

The UTAUT model also has moderating effects on the relationship between the constructs and the behavioral intention. The influence must be considered when calculating the model. A moderator is a qualitative or quantitative variable that influences the direction or strength of a relationship between an independent and a dependent variable [47]. In this case, gender, age and work experience are moderators that influence the relationships between the individual constructs and behavioral intention. In this work, MGA is very well suited to continuously investigate moderating effects throughout the model. For continuous facilitators, such as age and work experience, the use of the moderating effects in SmartPLS 3 is useful [14]. The PLS-MGA calculates two separate bootstraps for the groups to be compared. The results of the two bootstraps form the basis for the hypothesis review for the study of group differences. The parameter estimates from the bootstrap analysis can verify how likely differences exist between the groups. If there is a significant difference between the groups, it can be assumed that the group feature acts as a moderator in the model [48].

## 4. Results

### 4.1. Sample Description

A total of 239 fattening pig farmers completed the questionnaire. Only about 10% of the respondents were female. Thus, women are underrepresented in this study, since about a third of all employees in German agriculture are female [49]. The majority of the respondents live in North Rhine-Westphalia and Lower Saxony, where pig fattening is most concentrated in Germany [50]. About 20% of the respondents are older than 55 years, thus the average age of employees is slightly underrepresented, since a third of all agricultural employees in Germany is older than 55 years [49]. Two thirds of the respondents have an apprenticeship, were agricultural supervisors or visited a special agricultural college. In this case, the average of the educational distribution for German farmers, where 68% have the mentioned kinds of educational qualifications, is closely met [49]. Almost a third of the respondents do not participate in the IAW or other AWPs. The survey cannot be considered as representative, as the distribution in the criteria mentioned differs from the distribution of the population. Nevertheless, it is an interesting sample, especially with regard to the IAW participants as many of the participants are included. For further information on the respondents’ profiles see Appendix A.

### 4.2. Results of the PLS Analysis

For the identification and evaluation of the extended UTAUT model the PLS analysis was used. This method analyses the relationship between the latent variables of the UTAUT model. Latent constructs in the UTAUT model are not observable and are described by empirically collected indicators. To test the significance of the item loading, the bootstrap procedure was used with 5000 subsamples. All external loadings of the indicators exceed the required value of 0.6 and 0.7, respectively. The indicators that could remain in the model in course of examining indicator reliability and convergence criteria are performance expectancy, effort expectancy, social influence, facilitating conditions, hedonic motivation, price value, habit, experience with the IAW, risk awareness, trust and behavioral intention (see Appendix B).

To assess the reliability of the constructs, the composite reliability (CR) and the AVE were computed. All AVE and CR values are above the recommended threshold value, implying an acceptable construct reliability. Appendix C shows the results of the reliability of the constructs. To assess discriminant validity, the square root of the AVE must exceed the intercorrelation of the constructs with every other construct [39]. All square roots of AVE surpass the intercorrelations, thus supporting discriminant validity. All measures of discriminant validity meet the threshold value which is recommended in the literature (see Appendix D).

**Figure 2:**
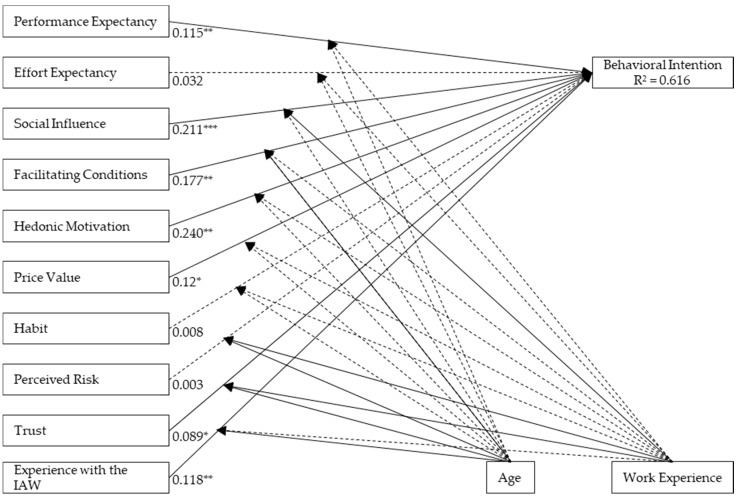
Results of the PLS analysis: Determinants of the participation in Initiative Animal Welfare (IAW). The values are presenting the path coefficients. Significance level: * *p* < 0.1, ** *p* < 0.05, *** *p* < 0.01; Broken line: not significant.

Figure 2 shows the results of the path coefficients calculated with the PLS algorithm. The paths of performance expectancy, social influence, facilitating conditions, hedonic motivation, price value, trust and experience with the IAW have a significantly positive influence on behavioral intention. Hedonic motivation and social influence have the strongest influence. The influence of trust is very small (0.089). According to [34], path coefficients below 0.1 are not truly interesting to interpret although it is significant. To examine the moderating effect of gender, age and work experience, MGA was used. Moderators can influence the strength and direction of a relationship in the model [47]. MGA is suitable because it allows to test a moderator continuously in the whole model [14]. The moderating effect of gender could not be verified due to a too small sample size of female respondents. This led to the problem of a singular matrix during the bootstrap procedure which is required for the MGA.

Age has a moderating effect on the relationship between experience with the IAW and behavioral intention, where the effect is stronger for older respondents (older than 43). The effect between trust and behavioral intention is also significantly stronger for older respondents. Work experience has a moderating effect on the relationship between social influence and behavioral intention and the relationship between trust and behavioral intention. The effect of trust is stronger for respondents with more work experience (more than 20 years). In contrast, the effect of social influence on behavioral intention is stronger for respondents with less work experience (see Appendix E). It becomes clear that 14 of the 20 hypotheses can be fully confirmed.

## 5. Discussion

Providing higher FAW standards is impossible without the acceptance of FAW programs by farmers. Therefore, we investigated the determinants ascertaining farmers’ intention to participate in the IAW in this study. It becomes clear that there is a relatively high willingness of farmers to participate. Nearly 70% of respondents intend to attend the IAW in the future. This is a much greater result than in other studies focused on this topic [51,52]. Statements such as “I have no interest in implementing higher animal welfare standards” are rejected by a vast majority of respondents. However, account must be taken of the fact that 69% of the respondents already participate in the IAW and thus the future willingness to participate is also increased. Ultimately, factors influencing the intention to participate were examined and not the participation itself. In course of the evaluation, several influencing factors were discernible. 

First of all, the hedonic motivation of the farmers has the strongest influence (0.240) on the intention to participate in the IAW and is, thus, the most important determinant for farmers’ participation in AWPs. Authors have repeatedly identified hedonic motivation as an important factor determining participation in AWPs [16]. In a study on hiring farmers for FAW, the farmer’s hedonic motivation, along with factors such as FAW and a good health system, has been identified as a determining factor for improving FAW in agriculture [16]. Without an intrinsic motivation of farmers to improve the housing conditions of pigs, implementation of FAWs is difficult [52]. In addition, it was found that a positive opinion about FAW labels significantly influences the willingness to participate in AWPs [18]. Almost all farmers mentioned that they enjoy bringing their pigs the best possible well-being. Furthermore, the results show that improving the image of pig fattening by participating in the IAW is an important motivation for many farmers. Further still, in another study, image improvement was seen as an important opportunity for participation in the IAW [51]. This finding also supports the results of a different study, which identified a group of farmers hoping to improve the image of pig farming by participating in AWPs [53].

As the second strongest influencing determinant, we found social influence (0.211). The factors influencing decisions of farmers in the context of adoption of innovations or systems have been widely studied in literature. Among other factors, the social environment plays a major role in many decisions made by farmers. Family, but also colleagues of the farmers influence their decisions [21,54]. It can be assumed that farmers discuss AIW participation with family members. The general attitude of farmers is also influenced by the social environment [20]. It has also been found that the social impact of the intention to participate in the IAW is greater among respondents with little work experience than those with more work experience. Inexperienced respondents, who may be still somewhat insecure in their professional practice, are more likely to be influenced by their private environment than more experienced farmers. The social impact does not only come from the farmer’s private environment. Thus, political or media pressure can also influence the intention to participate. The descriptive results show that farmers feel pressure to improve housing conditions at the farm, especially on the part of the media. For this reason, farmers hope for an image improvement of pig fattening by participating in the IAW.

Farmers’ perceptions of price value as well as farmers’ effort expectancy have been identified as influencing factors in this study. As mentioned before, farmers affirm that price value is very important to them. Nevertheless, the price value seems to be less important to the farmers than the hedonic motivation as well as the social influence. Basically, farmers are open to changes in the form of implementation of FAW criteria, as long as they are sufficiently remunerated [8]. Farmers willing to participate in AWPs require an increase in prices, often above what most consumers are willing to pay [18]. In this study, it can be seen that farmers usually do not consider the price value as sufficient. They feel that the time required and the stress they suffer, for example from unannounced audits, are not sufficiently remunerated. We find a positive correlation between price value and intention to participate (0.12). Nonetheless, farmers participate even if they do not feel equitably remunerated. Furthermore, in a study, the time required to implement FAW criteria was above all considered [54]. In another study, participants in AWPs saw the time-consuming burden of documentation and the time required for daily work in the stable [15].

Perceived risk seems to have a very low impact on farmers’ intention to participate in the IAW although participation in AWPs is perceived as an economic risk by some farmers [15,23]. Above all, the IAW’s unannounced control audits have increased the risk from the farmers’ perspective [29].

Facilitating conditions also have a positive influence on intention to participate in the IAW. It is likely that farmers who have good premises on their farms to implement FAW criteria of the IAW are more likely to participate. The statement “participation in the IAW results in deadweight effects” was confirmed on average by the respondents. The deadweight effect is an effect of the payment of financial incentives in which, even without additional incentive, the desired or partial change in behavior would have been present. The intention of the incentive to induce additional behavioral changes is therefore not fulfilled [55]. Other studies also confirmed that there were certain deadweight effects [8]. Others found that the higher additional expenses which have to be made to carry out the measures on the holding, the lower the willingness of farmers to participate [18]. Many farmers in the present study confirm that an implementation of the IAW is possible without any problems and they have the necessary framework for implementation. This is one of the IAW’s main approaches to implementing animal welfare without major structural changes in comparison to other AWPs. It becomes clear that many of the respondents do not have to make any major changes to the operation of the IAW’s animal welfare measures.

In case of the trust construct, a significant influence on the behavioral intention could also be demonstrated. However, the influence is very small (0.089). The distribution of responses to this construct makes it clear that farmers do not feel that they are being given sufficient consideration when it comes to setting wages, criteria to be met and documentation. However, the statement “I trust the IAE” is not rejected on average. It becomes clear that farmers want greater participation in the design of the program. This was also stated in a former study on farmers’ attitude towards AWPs [15]. Others found that measures such as additional space and organic manipulable materials are considered by farmers to be important and feasible [19]. It was also found that these measures are the most widely accepted by farmers, presumably, as some of the farmers can implement these measures without major structural measures on the farm [18]. This is probably one of the reasons why participation in the IAW is relatively high. The mandatory criteria for participation in the IAW are the provision of 10% more space and additional organic manipulable material. Farmers are able to implement measures that they consider acceptable themselves. For older respondents and the group with more work experience, trust has a greater influence on the behavioral intention. Presumably, trust plays a greater role in financial decisions, such as participating in an AWP, for older respondents who usually have more work experience. One explanation could be that trust decreases with age. For example, trust has a greater influence because older respondents are more distrustful than younger respondents [30].

Experience with the IAW also had a significant influence on the behavioral intention to participate in the IAW. Presumably, a positive or negative experience already made with the IAW or other AWPs will have an impact on the future behavioral intention to participate in the IAW. Older respondents were found to have more impact on the behavioral intention than younger respondents. This could be explained by the fact that older respondents have already gained more work experience and thus the influence on the behavioral intention is greater.

The question remains why the willingness to participate in IAW is so high. On the one hand, farmers can implement FAW criteria that they consider important and implementable, and on the other, many farmers already have the necessary framework conditions for their farms. Another reason why farmers generally support participation in the IAW could be consumer independence. Several previous studies have shown that many farmers question the willingness of consumers to pay more for FAW products. Moreover, the opinion is reflected that FAW products are still only a niche product and they fear that the economic benefits will be lost as a result of the lack of willingness to pay [15,23]. Since the IAW is financed by the food retailing industry and is therefore completely independent of consumers’ willingness to make a payment, this mistrust is not relevant when participating in the IAW. Many farmers also fear the IAW will eventually commit itself to the food retailers [8]. In particular, farmers are afraid that the IAW criteria will become a national standard and that they will not receive payments for providing additional FAW measures to their livestock. By joining early, farmers have the opportunity to get IAW’s investments balanced for FAW criteria. If the IAW commits itself in the future, farmers will no longer be paid to implement the criteria.

In principle, it is clear that farmers are willing to improve FAW. However, fair compensation is a prerequisite for participation. In addition, the hedonic motivation and, associated with that, the associated animal welfare awareness of the farmers plays a major role. Ultimately, the success of IAW depends on whether farmers are willing to volunteer in the program. In order to maintain the high level of willingness to participate in the future, an attempt should be made to influence the factors that make participation most likely. In order to influence motivation, it is important to analyze more closely what motivates farmers most. If, for example, they are interested in improving their image, IAW should try to make the consumer aware of IAW through increased publicity. IAW is already attempting to increase the transparency and awareness of FAW products by introducing the tiered labeling of meat products. The planned introduction of pork labels by IAW farms may further increase consumer awareness of the IAW [56]. In addition, as already mentioned, it becomes clear that farmers do not feel sufficiently taken into account in the design of the criteria as well as the documentation. Better involvement of farmers could increase farmers’ trust in the IAW. The high willingness to participate shows that the pig farmers are in favor for the concept. The involvement of farmers is an important aspect, which may enhance the willingness to participate in AWPs in the future. If the FAW criteria are accepted by farmers and considered important for the health and well-being of the animals, they are most likely to be more motivated to participate in AWPs [16]. Trust can thus also be increased. 

Conducting an online survey has a few limitations. It may limit the representativeness of the results across Germany. For example, there are differences in some groups in the use of the Internet. Women, people of older ages and those with less education use the Internet less frequently than men, younger people, and those with more education [57]. By conducting the survey online, some groups will be reached more easily than others. Thus, online surveys can have a so-called problem of representativeness. The representativeness can be improved by using a wide range of recruiting methods [58]. This was the case in the survey used for this study. In addition, the influence of the gender moderator could not be investigated. The reason for this is a too small sample of female respondents (*n* = 19). This led to the problem of a singular matrix. This influence can only be studied if the sample of female respondents is increased. By using the MGA to study the continuous moderators, only the differences of two groups could be investigated, suggesting a moderating effect. Some aspects could have got lost by this.

With regard to the question of the present work, the chosen model has a limitation. One limitation is that the actual use behavior could not be included in the model. The construct “use behavior” describes the actual participation in the IAW. As already explained in the Introduction, due to budget restrictions only about half of those farmers who wanted to participate were actually able to participate in the program. Thus, the authors assumed that these two groups of farmers did not differ with regard to their attitudes towards the IAW and their acceptance of the program. For this reason, the construct use behavior was excluded from the model. This means that only the influences on the intention to participate could be measured. Although this allows conclusions to be drawn about the actual behavior, it cannot be equated with it [59]. Thus, it could be that the actual participation is influenced by more factors, such as financial constraints or limited access to the program. There is also the possibility that not all farmers who indicate a willingness to participate would actually participate (attitude/behavior gap) [60]. Despite the limitations mentioned above, the UTAUT model seems to be the appropriate conceptual framework for the study being carried out. More than 60% of the variance of behavioral intention could be explained by the determinants included in the model. Furthermore, it is questionable whether an influence with a path coefficient smaller than 0.1 is worth considering. The guide values for path coefficients are discussed in literature. While some consider a value of 0.2 to be acceptable [34], others confirm a value of 0.1 [46]. The trust construct has a significant influence of 0.089 at a 10% level and was included in the model in the present work.

The interpretation of quality criteria regarding the model is diverse in scientific literature. Since all AVE and CR values are above the recommended threshold value, acceptable construct reliability can be implied. Some claim the threshold value of AVE is 0.5 [37]. The CR should ideally exceed a value of 0.6 [38]. Cronbach’s alpha describes the internal consistency of the construct while it should exceed a value of 0.7 [36]. It is also required to fulfil the HTMT criterion to reach discriminant validity [40]. The values of HTMT must not exceed a benchmark of 0.85. Furthermore, the cross loadings of the items must be examined. The loading on the corresponding construct must be higher on another construct [37]. The discriminant validity has been reached in this model.

## 6. Conclusions and Further Research

All in all, the study shows that the IAW is a functioning system well accepted by farmers. In addition to the acceptance of the farmers, there are two unique aspects of the IAW, which are important for farmers’ acceptance and which make the system interesting for other countries at least in the European Union. First, farmers receive a guaranteed and fixed sum for the higher FAW standards they provide. This increases their independence from consumer decisions at the point of sale where a gap between attitudes and actual buying behavior has been described [61]. Secondly, the FAW measures can be implemented by farmers without much effort, such as the construction of a new barn. Attendance figures from other European animal welfare programs, such as the Dutch label “Beter Leven”, “Coop Naturafarm” from Switzerland, or “Freedom food” from Great Britain indicate that the ease of implementing the required FAW measures is crucial for a broad acceptance among farmers. The IAW is thus a successful example of how the implementation of higher FAW standards can work in harmony with the interests of farmers and consumers. As this study identified important determinants of farmers’ participation in AWPs, it is relevant to explore how the individual determinants can be used and designed for farmers to ensure farmers’ willingness to participate internationally. However, so far, the existing international approaches are difficult to compare with the IAW, as they have little transparency regarding farmers’ remuneration and the criteria are partly inaccurately defined. Future studies could investigate these points in detail to receive a comprehensive international comparison between the different animal welfare programs that are actually on the market.

Furthermore, future research could cluster three groups of farmers and their attitudes as well as personal and farm characteristics. First, farmers who can and will participate; second, farmers who want to but cannot; and third, farmers who do not want to and do not participate. This analysis could lead to a further differentiation of the actual participants from those who only intend to participate or do not want to participate. This could thus help to design an even more tailor-made program. Furthermore, research on new solutions is needed to circumvent negatively perceived aspects of farmers participating in the IAW, such as anxiety and stress over unannounced audits and the amount of documentation required.

## Figures and Tables

**Table 1:** Applicable criteria in pig fattening in the IAW [5].

Basic and Mandatory Criteria	Remuneration
Basic criteria Quality and Safety (QS) Antibiotics monitoring by QS Slaughter animal evaluation by QS Stable climate check Drinking water check Daylight	€500 per year as a basic contribution for all expenses
Organic manipulable material Additional 10% space	Mandatory criteria
**Mandatory criteria in total**	**€3.30 per pig**
Additional 20% space	€1.20 per pig
Constant access to roughage	€1.80 per pig
Possibility to scrub	€0.60 per pig
Air cooling device	€0.20 per pig
Drinking from an open water source	€0.70 per pig
**Eligible criteria cannot exceed**	**€1.80 per pig**
**Maximum remuneration per pig**	**€5.10 per pig**

**Table 2:** Quality criteria with threshold values of reflective measurement models [37,40].

**Indicator Reliability**	Loadings > 0.7 [34]
	Cronbach’s alpha > 0.7 [36]
**Convergence Criteria**	AVE > 0.5 [37]
	Construct reliability > 0.6 [38]
**Discriminatory Validity**	Fornell-Larcker criterion > AVE [39]
	Cross loadings < loadings on the associated construct [37]
	HTMT of the correlations < 0.85 [40]

AVE, average variance extracted; HTMT, heterotrait/monotrait ratio.

## Appendix A

**Table A1:** Profile of the respondents, *n* = 239.

Variable	Description	Frequency	Percentage (%)
Gender, *n* = 239	Male	220	92.1
	Female	19	7.9
Age, *n* = 236	Under 25	10	4.2
	26–35	54	22.9
	36–45	56	23.7
	46–55	68	28.8
	56–65	45	19.1
	Older than 65	3	1.3
Agricultural Education, *n* = 239	Agricultural apprenticeship	10	4.2
	Agricultural “Meister”	68	28.5
	Agricultural college	48	20.1
	University degree	81	33.9
	Doctor degree	5	2.1
	other	26	10.9
	No graduation	1	0.4
German state, *n* = 239	Lower Saxony	73	30.5
	North Rhine-Westphalia	77	32.2
	Baden-Württemberg	26	10.9
	Bavaria	24	10
	Schleswig-Holstein	14	5.9
	other	25	1.5
Work experience, *n* = 156	Less than 10 years	32	20.5
	10 to 20 years	44	28.2
	21 to 30 years	36	23.1
	More than 30 years	44	28.2
Participation in IAW, *n* = 239	Yes	165	69
	no	74	31

## Appendix B

**Table A2:** Constructs with corresponding items, item loadings and descriptive analysis with a five-point Likert scale from 2 (totally agree) to −2 (totally disagree), *n* = 239.

Construct	Corresponding Item	Loading	Mean	SD
Performance Expectancy	PE1: Participation gives me a better conscience towards the animals	0.812	0.07	1.173
	PE2: Participation improves the welfare of the animals	0.925	0.5	0.958
	PE3: Participation improves the housing conditions of the animals	0.903	0.6	0.958
	PE4: Participation improves the health of the animals	0.857	0.18	1.069
Effort Expectancy	Participation is associated… EE1: …with high investment costs	0.696	0.68	0.965
	EE2: …with high labour costs	0.84	0.31	1.11
	EE3: …with high extra costs for certification	0.61	0.69	0.981
	EE4: …with high workload for daily work in the stable	0.844	0.51	1.086
	EE5: …with workload for documentation	0.601	1.05	0.915
	EE6: …with time-consuming controls	0.708	0.98	0.938
	EE7: …with high risks	0.695	0.24	1.23
	EE8: …with extra stress due to unannounced controls	0.628	0.93	1.106
Social influence	SI1: My colleagues support the participation	0.615	0.26	0.866
	SI2: My employees support the participation	0.825	0.22	1.008
	SI3: My family supports the participation	0.895	0.69	1.086
	SI4: My neighbours support the participation	0.626	0.28	1.109
	SI5: My purchasers support the participation	0.778	0.38	1.033
Facilitating conditions	FC1: On my farm I have the necessary framework conditions to participate	0.868	0.95	1.074
	FC2: The participation is uncomplicated for my farm	0.862	0.44	1.194
	FC3: Participation results in deadweight effects for my farm	0.737	0.56	1.124
Hedonic Motivation	HM1: I want to participate, because it is fun to try various animal welfare measures in the stable	0.773	1.34	0.762
	HM2: I want to participate to improve the image of conventional pig husbandry	0.877	0.91	1.065
	HM3: I want to participate to improve my reputation with people who are important to me	0.799	0.05	1.247
Price value	PV1: The additional workload is appropriately remunerated	0.887	−0.22	1.041
	PV2: The additional stress is appropriately remunerated	0.799	−0.5	0.984
	PV3: The animal welfare measures are appropriately remunerated	0.847	−0.23	1.014
	PV4: The participation is characterized by a good cost-benefit ratio	0.906	−0.3	0.979
	PV5: The participation pays off financially	0.875	−0.14	1.04
Habit	H1: The implementation of the animal welfare measures quickly becomes a habit	0.853	0.66	0.915
	H2: The controls by IAW quickly become a habit	0.792	−0.18	1.115
	H3: The documentation for IAW quickly become a habit	0.794	−0.09	1.077
	H4: The daily work processes on the farm hardly change due to Participation	0.726	0.17	1.089
Experience with IAW	E1: The controls by IAW are fair	0.797	0.53	0.894
	E2: The documentation for IAW is easy to understand	0.771	0.16	0.909
	E3: The accounting with IAW is uncomplicated	0.765	0.29	1.129
Risk awareness	RA1: Participation is associated with a financial risk	0.875	0.05	1.211
	RA2: Participation is associated with stress due to unannounced controls	0.676	0.88	1.047
	RA3: Participation is associated with longer working hours to implement the animal welfares measures	0.81	0.73	1.04
	RA4: Participation could bring the farm in a situation threatening its existence if a control is not passed	0.638	−0.29	1.144
Trust	The IAW makes effort to consider the needs and wishes of farmers… T1: …when designing the program	0.814	−0.21	0.897
	T2: …when designing the animal welfare measures	0.856	−0.34	0.922
	T3: …with the documentation	0.819	−0.38	0.918
	T4: I trust the IAW	0.835	0.25	0.984
Behavioural Intention	BI1: It makes sense to participate in IAW	0.936	0.78	1.062
	BI2: I intend to participate in IAW in the future	0.919	0.85	1.217

## Appendix C

**Table A3:** Internal consistency: Cronbach’s alpha, composite reliability and average variance extracted of the constructs.

Construct	Cronbach’s Alpha	Composite Reliability (CR)	Average Variance Extracted (AVE)
Performance Expectancy	0.897	0.929	0.766
Effort Expectancy	0.868	0.891	0.508
Social Influence	0.81	0.873	0.572
Facilitating Conditions	0.763	0.864	0.68
Hedonic Motivation	0.763	0.85	0.654
Price Value	0.915	0.936	0.746
Habit	0.803	0.871	0.628
Perceived Risk	0.76	0.84	0.572
Trust	0.857	0.912	0.691
Experience with IAW	0.674	0.821	0.605
Behavioural Intention	0.838	0.925	0.86

## Appendix D

**Table A4:** Discriminant validity: Fornell–Larcker criterion.

Construct	BI	EE	E	FC	H	HM	PE	PV	PR	SI	T
**BI**	**0.927**										
**EE**	−0.196	**0.713**									
**E**	0.49	−0.335	**0.778**								
**FC**	0.509	−0.241	0.361	**0.824**							
**H**	0.506	−0.502	0.542	0.512	**0.792**						
**HM**	0.628	−0.076	0.327	0.352	0.397	**0.809**					
**PE**	0.588	−0.087	0.355	0.283	0.383	0.651	**0.875**				
**PV**	0.56	−0.452	0.539	0.391	0.579	0.425	0.452	**0.864**			
**PR**	−0.29	0.643	−0.339	−0.31	−0.529	−0.158	−0.186	−0.488	**0.756**		
**SI**	0.663	−0.207	0.413	0.464	0.514	0.595	0.635	0.555	−0.345	**0.756**	
**T**	0.53	−0.213	0.517	0.376	0.512	0.411	0.429	0.552	−0.284	0.493	**0.831**

Highlighted: square root of average variance extracted (AVE). BI: Behavioral Intention; EE: Effort Expectancy; E: Experience with IAW; FC: Facilitating Conditions; H: Habit; HM: Hedonic Motivation; PE: Performance Expectancy; PV: Price Value; PR: Perceived Risk; SI: Social Influence; T: Trust.

## Appendix E

**Table A5:** Results of multigroup analysis: the moderating effect of age and work experience between the constructs and behavioral intention.

	Path Coefficients Age			Path Coefficients Work Experience		
Path	Younger than 45	Older than 43	Difference	Less than 21 Years	More than 20 Years	Difference
**EE -> BI**	0.032	0.026	0.005	−0.016	−0.004	0.012
**E -> BI**	−0.01	0.192	0.202 *	−0.016	0.08	0.096
**FC -> BI**	0.18	0.197	0.017	0.162	0.245	0.083
**H -> BI**	0.056	−0.03	0.087	0.155	−0.081	0.236
**HM -> BI**	0.225	0.268	0.043	0.205	0.319	0.114
**PE -> BI**	0.171	0.006	0.165	0.132	0.114	0.019
**PV -> BI**	0.208	0.028	0.179	0.028	−0.015	0.043
**PR -> BI**	0.053	−0.01	0.063	0.055	−0.014	0.07
**SI -> BI**	0.271	0.207	0.064	0.423	0.082	0.341 **
**T -> BI**	−0.031	0.199	0.23 **	−0.059	−0.338	0.397 **

Significance level: ** *p* < 0.05, * *p* < 0.1. BI: Behavioural Intention; EE: Effort Expectancy; E: Experience with IAW; FC: Facilitating Conditions; H: Habit; HM: Hedonic Motivation; PE: Performance Expectancy; PV: Price Value; PR: Perceived Risk; SI: Social Influence; T: Trust.
